# The Complete Chloroplast Genome of the Hare’s Ear Root, *Bupleurum falcatum*: Its Molecular Features

**DOI:** 10.3390/genes7050020

**Published:** 2016-05-13

**Authors:** Dong-Ho Shin, Jeong-Hoon Lee, Sang-Ho Kang, Byung-Ohg Ahn, Chang-Kug Kim

**Affiliations:** 1 Genomics Division, National Institute of Agricultural Sciences, Jeonju 54874, Korea; dhshin013@korea.kr (D.-H.S.); hosang93@korea.kr (S.-H.K.); 2 Department of Herbal Crop Research, National Institute of Horicultural and Herbal Science, Eumseong 55365, Korea; artemisia@korea.kr; 3 Research Policy Planing Division, Rural Development Administration, Jeonju 54875, Korea; boahn@korea.kr

**Keywords:** *Bupleurum falcatum*, chloroplast genome, quadripartite structure, tandem repeats, phylogenetic analysis

## Abstract

*Bupleurum falcatum,* which belongs to the family *Apiaceae,* has long been applied for curative treatments, especially as a liver tonic, in herbal medicine. The chloroplast (cp) genome has been an ideal model to perform the evolutionary and comparative studies because of its highly conserved features and simple structure. The *Apiaceae* family is taxonomically close to the *Araliaceae* family and there have been numerous complete chloroplast genome sequences reported in the *Araliaceae* family, while little is known about the *Apiaceae* family. In this study, the complete sequence of the *B. falcatum* chloroplast genome was obtained. The full-length of the cp genome is 155,989 nucleotides with a 37.66% overall guanine-cytosine (GC) content and shows a quadripartite structure composed of three nomenclatural regions: a large single-copy (LSC) region, a small single-copy (SSC) region, and a pair of inverted repeat (IR) regions. The genome occupancy is 85,912-bp, 17,517-bp, and 26,280-bp for LSC, SSC, and IR, respectively. *B. falcatum* was shown to contain 111 unique genes (78 for protein-coding, 29 for tRNAs, and four for rRNAs, respectively) on its chloroplast genome. Genic comparison found that *B. falcatum* has no pseudogenes and has two gene losses, *accD* in the LSC and *ycf15* in the IRs. A total of 55 unique tandem repeat sequences were detected in the *B. falcatum* cp genome. This report is the first to describe the complete chloroplast genome sequence in *B. falcatum* and will open up further avenues of research to understand the evolutionary panorama and the chloroplast genome conformation in related plant species.

## 1. Introduction

Chloroplasts, also called cp or ct, are distinctly important organelles which have their own genomes and play pivotal roles in generating energy through photosynthesis, fixing carbon, and biosynthesizing starch, fatty acids, pigments, and amino acids in plant cells [1,2]. They have been used as ideal research models, particularly for evolutionary and comparative genomic studies because of their highly conserved gene content, relatively small size, and simple structure. 

A complete chloroplast genome sequence was first reported from tobacco, *Nicotiana tabacum,* in 1986 [3] after the existence of the chloroplast DNA was ultrastructurally confirmed from *Chlamydomonas* in 1962 [4]. To date, hundreds of complete chloroplast genome sequences have been identified from plants, algae and bacteria. Plant chloroplasts have been known to be derived from the endosymbiosis of cyanobacteria and their genomes have been reported to inherit uniparentally without recombination, although some plants have been reported to biparentally inherit [5,6]. The chloroplast genome can be divided into two comprehensive categories including protein-coding genes and non-coding regions; the latter is further divided into introns and intergenic regions [7]. The genome is unified in a conserved quadripartite structure consisting of a large single copy (LSC), a small single copy (SSC), and a set of mutually-inverted repeat sequences (IRa and IRb) [8,9,10], which are also highly conserved together with gene order and content among species [11]. Therefore, comparative studies of the chloroplast genome have been used not only to solve the jigsaw puzzle of plant evolutionary history but also to identify taxonomical position [12,13,14], although large-scale genome rearrangement and gene loss have been reported in several angiosperm lineages [15,16]. Information from the chloroplast genome is beneficial to phylogenetics [17], DNA barcoding [18], population biology [19], and transcriptomic [20] studies. It is important to understand how plant species are linked, what features are shared among them, and how they are different from other taxonomic groups. Sequencing the complete chloroplast genome is now inexpensive and efficient using next generation sequencing (NGS) technology. 

In this study, the complete chloroplast genome of a flowering plant, *Bupleurum falcatum,* which is categorized to the family *Apiaceae,* was investigated. The *Apiaceae* family is taxonomically close to the *Araliaceae* family and there have been numerous complete chloroplast genome sequences reported in the *Araliaceae* family [21,22], while little is known about the *Apiaceae* family. *B. falcatum* (known as Chinese thoroughwax and Sickle hare’s ear, called Siho in Korean) is a perennial plant and has long been known to be a medicinal plant because it produces curative compounds that have been reported to possess various beneficial properties [23,24,25,26], especially as a liver tonic, in herbal medicine. This report is the first to describe the complete chloroplast genome sequence, sequence analysis, and molecular and phylogenetic comparison of *Bupleurum falcatum* belonging to the *Apiaceae* family. It is believed that the results may contribute to a better understanding of genetic relationships and evolutionary aspects of medicinal plants in the families *Apiaceae* and *Araliaceae*.

## 2. Results and Discussion

### 2.1. Sequencing, Assembly, and Validation of the B. falcatum Chloroplast Genome

**Figure 1:**
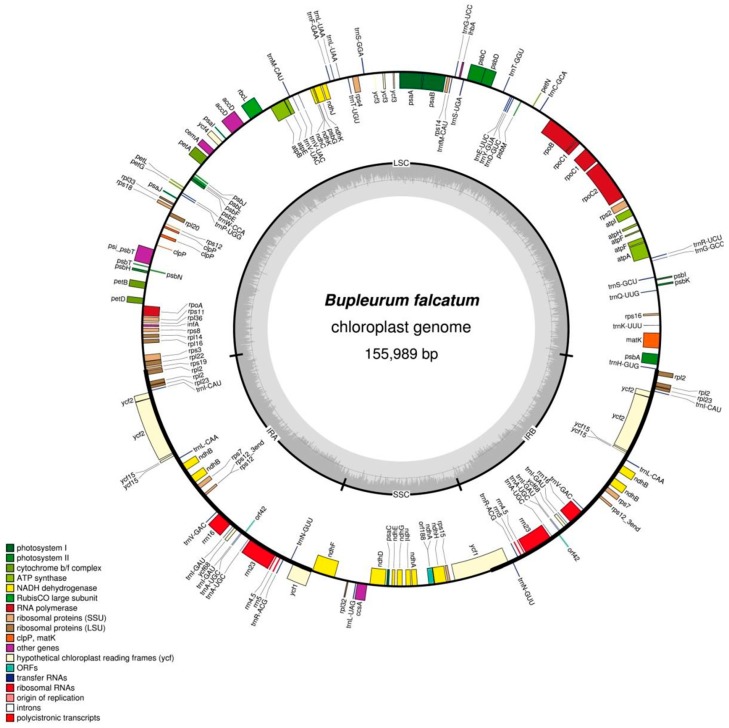
The gene map of the chloroplast genome of *Bupleurum falcatum*. The Organellar Genome Draw (OGDraw) program was applied for drawing the map. LSC, SSC, and IR are abbreviated for large single copy, small single copy, and inverted repeat, respectively. Genes located inside and outside of the outer rim are transcribed clockwise and counterclockwise, respectively. Functionally-annotated genes are seen in colored portions. The dashed gray area in the inner circle shows the proportional GC content of the corresponding genes.

A total of eight million paired-end (PE) reads (800 million nucleotides) generated were trimmed and assembled using a CLC genome assembler (CLC Bio, Aarhus, Denmark). A total of 342,316 PE reads were concordantly mapped to the final assembly with an estimation of the cp coverage (194.83×). The mapped cp contigs were selected and merged to construct a complete *B.falcatum* cp genome using a MUMmer (Hamburg University, Hamburg, Germany). The *B. falcatum* cp genome sequence was generated from a combined product of four initial contigs; contigs 8, 27, 76, and 209, respectively with no gaps and no Ns. The assembled *B. falcatum* cp genome showed a high similarity to the reference sequence, *Panax ginseng* Chunpoong (GenBank Accession#: KM088019) and extended its size to 155,989 nucleotides long (Figure 1). The cp genome sequence was registered into the DNA Data Bank of Japan (DDBJ).

European Molecular Biology Laboratory (EMBL), and GenBank with the accession number KC207676. The largest and smallest assembled sequences were derived from contigs 8 and 209 covering the complete regions of the LSC and SSC, respectively. As shown in Figure 1, the *B. falcatum* cp genome is a typical quadripartite structure composed of the LSC, SSC and a pair of IR regions, indicating that the assembled *B. falcatum* cp genome sequence represented full coverage with no abnormalities. *B. falcatum* belongs to the *Apiaceae* family in plant classification. Recently, several chloroplast genome sequences have been reported from the *Apiaceae* and *Araliaceae* families such as *P. ginseng*, *A. undulate, D. carota and A. cerefolium* [21,22,27]. Both *Apiaceae* and *Araliaceae* families belong to the *Apiales* order and these two families are known to have the most abundant species ranked into the largest and second, respectively, in the order [28]. The overall sequencing results of the *B. falcatum* cp genome compared with genome size and physical shape indicate that there are no significant differences between *B. falcatum* chloroplast genome and those from the two closely-related families. 

### 2.2. Physical Features of the B. falcatum Chloroplast Genome

The complete *B. falcatum* cp genome obtained in this study appears to be a typical circular form and encodes 155,989 nucleotides encompassed in the quardripartite structure which is built in four regions (LSC, SSC, IRa and IRb) described elsewhere [3,29] (Figure 1). The respective four regions of the cp genome occupy 85,912 bp for LSC, 17,517 bp for SSC, and 52,560 bp (26,280 bp each) for a set of the IR regions, accounting for about 55.1% for LSC, 11.2% for SSC and 33.7% for IRs, respectively (Table 1). Table 2 describes a list of the corresponding genes according to their functions, features and participational roles.

The *B. falcatum* cp genome contains a total of 111 unique genes constituting 78 protein-coding genes, 29 transfer RNAs, and four ribosomal RNAs with the overall AT content of 62.34% (Table 1). Seventeen genes are identified to contain at least one intron and two of them (*clpP and ycf3*) are shown to contain two introns (Table 1 and Table 2). Among the genes containing intron(s), twelve genes appear to belong to the group of the protein-coding genes and five to tRNAs (Table 2). For the nucleotide occupancy, 88,065 bp of the nucleotide length accounts for the genic regions which contain both exonic and intronic portions corresponding to 56.46% and 67,924 bp for intergenic regions corresponding to 43.54%, respectively (Table 1).

The gene composition of the four respective regions in the *B. falcatum* cp genome shows that the LSC region contains 60 genes for the protein-codings and 21 for tRNAs (Figure 1). The cp genome also shows that the SSC region encodes 11 genes for the protein-codings and one for tRNA. Furthermore, it shows that respective IR regions express five genes for the protein-codings, seven for tRNAs, four for rRNAs, and that the border junctions between the SC regions and the IR regions contain two protein-coding genes. As shown in this result, all of the ribosomal RNAs of the *B. falcatum* chloroplast genome are housed only in the IR regions, suggesting that the IRs play a critical role in the existence of the chloroplast in *B. falcatum* as well as in other plant species. It is also speculated that the IR regions have been a very conserved partitive wall to specify ribosomal RNAs for the ribosome build-up through the long-term evolutionary events. 

Moreover, comparative analysis of gene content, interestingly, shows that there are unusual features on the *B. falcatum* cp genome (Supplementary Table S1). There are two gene losses of *accD* and *ycf15* detected in the *B. falcatum* cp genome.As compared in Supplementary Table S1, the *accD* gene is usually located between the *rbcL* and *psaI* *genes* in the LSC region, and the *ycf15* gene duplicates at the position between *ycf2* and *trnL-CAA* in the IR regions among the compared plant species of *Araliaceae* and *Apiaceae* families. In addition, a gene substitution of *lhbA* is recognized at the position where the *psbZ* gene has generally been shown at the position between the genes *trnS* and *trnG* in related species [22,27]. The *accD* gene encodes a subunit of heteromeric acetyl-CoA carboxylase (ACCase) that is commonly found in plastids of dicots but is not seen in the rice and other *Gramineae* [30]. It will be required to confirm whether the two genes *accD* and *ycf15* have been completely missing from the chloroplast genome of *B. falcatum* or have been transferred to other cellular organelles such as the nucleus, although there have been reports with respect to no *ycf15* gene (GenBank accession number, GU456628) and the *accD* gene’s loss [31,32,33]. 

Some of the genes in the chloroplast genome have been reported to pseudogenize, particularly genes in the IR regions of plant species. Plant species featuring pseudogenization have recently been reported from the *Apiales* order including *A. cerefolium*, *Panax ginseng*, *A. carota*, *B. hainla*, and *K. septemlobus* [21,22]. However, there were no pseudogenes identified in the *B. falcatum* cp genome in this study (Supplementary Table S1). It is not clear if *B. falcatum* has a specific kinetic mechanism different from the other species. Future investigation will be required to elucidate what mechanism conducts the pseudogenization.

### 2.3. Tandem Repeat Sequences

The repeated sequences come in various sizes in the pattern and are classified according to the length of the core repeat units, the number of contiguous repeat units, and/or the overall length of the repeat region. In order to investigate simple sequence repeats in *B. falcatum*, tandem repeats (TRs) were analyzed. A total of 54 unique consensus sequences (out of 119 copies) of tandem repeats were detected at 55 locations (consensus 16 and 17 are identical but occur at different positions) from the *B. falcatum* chloroplast genome DNA (Supplementary Table S2) using Tandem Repeat Finder, version 4.0 (LBI, Boston University, Boston, MA, USA) [34]. Out of them, 53 repeat locations appear to be arranged by two copies, while one position appears to be arranged by three copies at nucleotides (nt) 49,794–49,811, and the remaining one by ten copies at nt 85,644–85,663. The shortest and longest tandem repeat sequences are identified to be one di- and one pentacontakai-penta-nucleotide repeats, which are positioned at nt 85,644–85,663 and nt 32,737–32,846, respectively. By length, 13 different sizes of the TR sequences ranging from 2 to 55 nucleotides reside in the *B. falcatum* chloroplast genome. The most abundant repeats lengths are found to be an octa-nucleotide and a nona-nucleotide, which occur at twelve and thirteen locations, respectively. There are seven single locations of tandem repeats found in *B. falcatum* as follows: di-, deca-, tetradeca-, hexadeca-, heptadeca-, nonadeca-, and pentacontakai-penta-nucleotide sequences, respectively. Considering the regional occupancy of the TR sequences, the IR regions have remarkably low frequency of the tandem repeats, showing only four locations corresponding to 7.02% compared to 42 and seven positions corresponding to 73.68% and 12.28% for the LSC and the SSC, respectively. Interestingly, most of the tandem repeats observed in the *B. falcatum* cp genome are identified to be located in the noncoding regions, whereas only a small portion of them is found to be in the genic regions of the following: *psaI*, *rpoA*, *rpl22*, *ycf2, ndhF, ndhD,* and *ndhI*. Recent reports have described the existence of five and eight protein-coding genes containing the repeat sequences, respectively, in *A. undulate* [22] and *P. ginseng Damaya* [27]. Many reports have mentioned a wide variety of repeat sequences across different plant species [3,21,22], albeit the loci and the genes are quite different. 

The results in this study indicate that the LSC region is relatively active in causing sequence variations, while the IR regions have much less kinetic pressure, suggesting that the conservation of the IR regions may be pivotally important to maintaining the functional integrity of plant chloroplasts including *B. falcatum*.

## 3. Experimental Section

### 3.1. Plant and DNA Sample Preparation

Based on its clinical importance in herbal medicine, *B. falcatum* was employed for the study. The plant was maintained in an indoor facility with an automatically and environmentally controlled system at the National Institute of Horicultural and Herbal Science, Rural Development Administration in Eumseong, Korea. The total plant DNA was extracted from the young leaves of *B. falcatum* using the modified cetyltrimethylammonium bromide (CTAB) method [35]. The entire process of DNA sample preparation for the whole genome shotgun sequencing was performed according to the manufacturer’s instructions of the Illumina platform (San Diego, CA, USA).

### 3.2. Chloroplast Genome Sequencing, Assembly, and Validation

The *B. falcatum* Illumina PE library was sequenced on a Hiseq 1000 genome analyzer platform (Illumina, San Diego, CA, USA) installed in the Genome Sequencing Center, the Department of Biotechnology, National Academy of Agricultural Science, Rural Development Administration in Suwon, South Korea. Low quality sequences (Phred score < 20) were trimmed and the remaining high quality sequences were assembled in to contigs using a CLC genome assembler beta 4.06 (CLC Inc., Rarhus, Denmark) with parameters of a minimum of 150–500 bp autonomously controlled overlap size at Phyzen Inc. (Seoul, South Korea). The principal contigs representing the chloroplast genome were obtained from the total assembled contigs using MUMmer [36] with the cp genome sequence of *Panax ginseng* cv. ChunPoong (KM088019) [37] as a reference sequence. The obtained chloroplast sequence contigs were ordered and oriented based on the previously reported cp genome sequence, and a complete chloroplast sequence was constructed by connecting overlapping terminal sequences. Validation was performed using the BlastZ program (Miller Lab, Penn State University, University Park, PA, USA) by comparing the structure of the *B. falcatum* cp genome with published cp genomes registered in National Center for Biotechnology Information (NCBI).

### 3.3. Chloroplast Genome Annotation, Codon Usage and Repeat Sequence Analysis

In order to annotate the chloroplast genome, the program DOGMA (Jansen Lab, UT Austin, TX, USA) [38] equipped with manually-operated corrections for start and stop codons was employed. The tRNA genes were identified using DOGMA and tRNAscanSE (Lowe Lab, UCSC University, Santa cruz, CA, USA) [39]. The cp genome map was constructed based on the program OGDraw (http://ogdraw.mpimp-golm.mpg.de/) [40]. Repeat sequences of the *B. falcatum* cp genome were assessed using Tandem repeat finder, version 4.0 (LBI, Boston University, Boston, MA, USA) [34] and REPuter (Faculty of Technology, Bielefeld University, Bielefeld, Germany) [41] with the following parameters: Match 2, Mismatch 7, Delta 7, PM 80, PI 10, Minscore 50, MaxPeriod 500, with similarity 100%, respectively.

## 4. Conclusions

There has been no report of the complete cp genome sequence of the *B. falcatum* species. We sequenced the complete *B. falcatum* chloroplast genome. The size, structure, gene content, and the compositional organization are not significantly different from most of the other chloroplast genomes reported from closely-related plant species, albeit there are subtle differences in features. It is a noticeable finding that there are no pseudogene(s), particularly in the boundary areas of the IR regions of the *B. falcatum* chloroplast genome. In addition, the *B. falcatum* cp genome showed the losses of *accD* and *ycf15* genes. However, further investigations of pseudogene(s) and the two missing genes remain to be performed. Future comparative analyses will be paid to elucidate how *B. falcatum* has evolved and diverged within the *Araliaceae* family. This report will open up further avenues of research to understand the genomic information and gene contents of the chloroplasts of the genus *Bupleurum* and the related families.

## Figures and Tables

**Table 1:** Summary of *de novo* assembly and general features of the chloroplast genome of *B.* *falcatum*.

Compositional Category	Features in Chloroplast
CP genome length (nt)	155,989
LSC length (nt)	85,912
SSC length (nt)	17,517
IR length (Ira/b) (nt)	52,560
AT/GC contents (%)	62.34/37.66
No. of total/unique genes	129/111
Genic occupancy (nt)	88,065
Intergenic occupancy (nt)	67,924
Protein-coding genes	78
Transfer RNAs (tRNAs)	29
Ribosomal RNAs (rRNAs)	4
No. of genes duplicated in IR regions	16
Total number of genes with intron(s)	17
Gene(s) with single intron	14
Gene(s) with multiple introns	3
tRNAs with intron(s)	5

nt indicates the units of nucleotides

**Table 2:** Gene composition in *B. falcatum* chloroplast genome (total 111 unique genes).

Category of Gene Group	Group of Genes	Name of Genes
Self replication	Ribosomal RNAs	16S (*rrn16)(x2),* 23S *(rrn23)(x2)*
		4.5S *(rrn4.5)(x2),* 5S *(rrn5)(x2)*
		*trnH-GUG, trnK-UUU*, *trnQ-UUG*
		*trnS-GCU,* *trnG-UCC* *^†^, trnR-UCU*
		*trnC-GCA, trnD-GUC, trnY-GUA*
		*trnE-UUC, trnT-GGU, trnS-UGA*
		*trnfM-CAU, trnS-GGA, trnT-UGU*
		*trnL-UAA ^†^, trnF-GAA, trnV-UAC ^†^*
	Transfer RNAs	*trnM-CAU, trnW-CCA, trnP-UGG, trnI-CAU(x2), trnL-CAA(x2), trnV-GAC(x2), trnI-GAU(x2) ^†^, trnA-UGC(x2) ^†^, trnR-ACG(x2), trnN-GUU(x2), trnL-UAG*
	Small subunit of ribosome	*rps2, rps3, rps4, rps7(x2), rps8, rps11, rps12(x2, part) ^†^, rps14, rps15, rps16 ^†^, rps18, rps19*
	Large subunit of ribosome	*rpl2(x2) ^†^, rpl14, rpl16 ^†^, rpl20, rpl22, rpl23(x2), rpl32, rpl33, rpl36*
	RNA polymerase	*rpoA, rpoB, rpoC1 ^†^, rpoC2*
Photosynthesis	NADH-	*ndhA ^†^, ndhB(x2) ^†^, ndhC, ndhD, ndhE*
	dehydrogenase	*ndhF, ndhG, ndhH, ndhI, ndhJ, ndhK*
	Photosystem I	*psaA, psaB, psaC, psaI, psaJ, ycf3 ^#^,*
	Photosystem II	*lhbA*, *psbA, psbC, psbD, psbE, psbF*
		*psbH, psbI, psbJ, psbK, psbL, psbM*
		*psbN, psi-psbT, psbT*
	Cytochrome b/f	*petA, petB ^†^, petD ^†^, petG, petL, petN*
	ATP synthase	*atpA, atpB, atpE, atpF ^†^, atpH, atpI*
	Rubisco	*rbcL*
Other genes		*infA*, *matK*, *clpP* *^#^*, *cemA*, *ccsA*
Unknown function	ORFs ^¥^	*ycf1*(x2, part), *ycf2*(x2), *ycf4*

*^†^* indicates the existence of single intron in the corresponding genes; *^#^* indicates the existence of two introns in the corresponding genes; ^¥^ indicates open reading frame. Genes duplicated in the IR region are represented by the (×2).

## Supplementary Materials

The following are available online at: http://www.mdpi.com/2073-4425/7/5/20/s1, Table S1: A comparative list of genes present in the chloroplast genomes of *B. falcatum* and others of the Araliaceae and Apiaceae families, Table S2: The result of tandem repeat sequences detected in the *B. falcatum* chloroplast genome sequence.

## Abbreviations

The following abbreviations are used in this manuscript:

LSC: Large Sequence Copy
IR: Inverted Repeat
SSC: Small Sequence Copy
TR: Tandem Repeats
CP: Chloroplast
