# Supplementary material for: The Complete Chloroplast Genome of the Hare’s Ear Root, Bupleurum falcatum: Its Molecular Features
Source: Genes (Basel). 2016 May 13;7(5):20. doi: 10.3390/genes7050020 (PMC4880840; doi:10.3390/genes7050020)
Supplement: Supplementary file 1 [file genes-07-00020-s001.docx]

Supplementary Materials: The Complete Chloroplast Genome of the Hare’s Ear Root, *Bupleurum falcatum*: Its Molecular Features

Dong-Ho Shin, Jeong-Hoon Lee, Sang-Ho Kang, Byung-Ohg Ahn and Chang-Kug Kim

**Table S1.** A comparative list of genes expressed in the chloroplast genomes of *B. falcatum* and others of *Araliaceae* and *Apiaceae* families.

| **Orders** | **KC456163 ^￥^** | **KC456164 ^￥^** | **KC456165 ^￥^** | **KC456166 ^￥^** | **KC456167 ^￥^** | **KC686332 ^￥^** | **KC686333 ^￥^** | **AY582139 ^￥^** | **GU456628 ^￥^** | **JN637765 ^￥^** | **KC207676 ^￥^** |
| --- | --- | --- | --- | --- | --- | --- | --- | --- | --- | --- | --- |
| 1 | rps12 | rps12 | rps12 | rps12 | rps12 | rps12 | rps12 | rps12 | rps12 | rps12 | rps12 |
| 2 | trnH-GUG | trnH-GUG | trnH-GUG | trnH-GUG | trnH-GUG | trnH-GUG | trnH-GUG | trnH-GUG | trnH-GUG | trnH-GUG | trnH-GUG |
| 3 | psbA | psbA | psbA | psbA | psbA | psbA | psbA | psbA | psbA | psbA | psbA |
| 4 | trnK-UUU | trnK-UUU | trnK-UUU | trnK-UUU | trnK-UUU | trnK-UUU | trnK-UUU | trnK-UUU | trnK-UUU | trnK-UUU | trnK-UUU |
| 5 | matK | matK | matK | matK | matK | matK | matK | matK | matK | matK | matK |
| 6 | rps16 | rps16 | rps16 | rps16 | rps16 | rps16 |  | rps16 | rps16 | rps16 | rps16 |
| 7 | trnQ-UUG | trnQ-UUG | trnQ-UUG | trnQ-UUG | trnQ-UUG | trnQ-UUG | trnQ-UUG | trnQ-UUG | trnQ-UUG | trnQ-UUG | trnQ-UUG |
| 8 | psbK | psbK | psbK | psbK | psbK | psbK | psbK | psbK | psbK | psbK | psbK |
| 9 | psbI | psbI | psbI | psbI | psbI | psbI | psbI | psbI | psbI | psbI | psbI |
| 10 | trnS-GCU | trnS-GCU | trnS-GCU | trnS-GCU | trnS-GCU | trnS-GCU | trnS-GCU | trnS-GCU | trnS-GCU | trnS-GCU | trnS-GCU |
| 11 | trnG-UCC | trnG-UCC | trnG-UCC | trnG-UCC | trnG-UCC |  |  | trnG-UCC | *trnG-GCC* | trnG-UCC | trnG-UCC |
| 12 | trnR-UCU | trnR-UCU | trnR-UCU | trnR-UCU | trnR-UCU | trnR-UCU | trnR-UCU | trnR-UCU | trnR-UCU | trnR-UCU | trnR-UCU |
| 13 | atpA | atpA | atpA | atpA | atpA | atpA | atpA | atpA | atpA | atpA | atpA |
| 14 | atpF | atpF | atpF | atpF | atpF | atpF | atpF | atpF | atpF | atpF | atpF |
| 15 | atpH | atpH | atpH | atpH | atpH | atpH | atpH | atpH | atpH | atpH | atpH |
| 16 | atpI | atpI | atpI | atpI | atpI | atpI | atpI | atpI | atpI | atpI | atpI |
| 17 | rps2 | rps2 | rps2 | rps2 | rps2 | rps2 | rps2 | rps2 | rps2 | rps2 | rps2 |
| 18 | rpoC2 | rpoC2 | rpoC2 | rpoC2 | rpoC2 | rpoC2 | rpoC2 | rpoC2 | rpoC2 | rpoC2 | rpoC2 |
| 19 | rpoC1 | rpoC1 | rpoC1 | rpoC1 | rpoC1 | rpoC1 | rpoC1 | rpoC1 | rpoC1 | rpoC1 | rpoC1 |
| 20 | rpoB | rpoB | rpoB | rpoB | rpoB | rpoB | rpoB | rpoB | rpoB | rpoB | rpoB |
| 21 | trnC-GCA | trnC-GCA | trnC-GCA | trnC-GCA | trnC-GCA | trnC-GCA | trnC-GCA | trnC-GCA | trnC-GCA | trnC-GCA | trnC-GCA |
| 22 | petN | petN | petN | petN | petN | petN | petN | petN | petN | petN | petN |
| 23 | psbM | psbM | psbM | psbM | psbM | psbM | psbM | psbM | psbM | psbM | psbM |
| 24 | trnD-GUC | trnD-GUC | trnD-GUC | trnD-GUC | trnD-GUC | trnD-GUC | trnD-GUC | trnD-GUC | trnD-GUC | trnD-GUC | trnD-GUC |
| 25 | trnY-GUA | trnY-GUA | trnY-GUA | trnY-GUA | trnY-GUA | trnY-GUA | trnY-GUA | trnY-GUA | trnY-GUA | trnY-GUA | trnY-GUA |
| 26 | trnE-UUC | trnE-UUC | rnE-UUC | trnE-UUC | trnE-UUC | trnE-UUC | trnE-UUC | trnE-UUC | trnE-UUC | trnE-UUC | trnE-UUC |
| 27 | trnT-GGU | trnT-GGU | trnT-GGU | trnT-GGU | trnT-GGU | trnT-GGU | trnT-GGU | trnT-GGU | trnT-GGU | trnT-GGU | trnT-GGU |
| 28 | psbD | psbD | psbD | psbD | psbD | psbD | psbD | psbD | psbD | psbD | psbD |
| 29 | psbC | psbC | psbC | psbC | psbC | psbC | psbC | psbC | psbC | psbC | psbC |
| 30 | trnS-UGA | trnS-UGA | trnS-UGA | trnS-UGA | trnS-UGA | trnS-UGA | trnS-UGA | trnS-UGA | trnS-UGA | trnS-UGA | trnS-UGA |
| 31 | psbZ | psbZ | psbZ | psbZ | psbZ | *lhbA* | *lhbA* | *ycf9* | psbZ | psbZ | *lhbA** |
| 32 | trnG-GCC | trnG-GCC | trnG-GCC | trnG-GCC | trnG-GCC | trnG-UCC | trnG-UCC | trnG-GCC | trnG-UCC | trnG-GCC | trnG-UCC |
| 33 | trnfM-CAU | trnfM-CAU | trnfM-CAU | trnfM-CAU | trnfM-CAU | trnfM-CAU | trnfM-CAU | trnfM-CAU | trnfM-CAU | trnfM-CAU | trnfM-CAU |
| 34 | rps14 | rps14 | rps14 | rps14 | rps14 | rps14 | rps14 | rps14 | rps14 | rps14 | rps14 |
| 35 | psaB | psaB | psaB | psaB | psaB | psaB | psaB | psaB | psaB | psaB | psaB |
| 36 | psaA | psaA | psaA | psaA | psaA | psaA | psaA | psaA | psaA | psaA | psaA |
| 37 | ycf3 | ycf3 | ycf3 | ycf3 | ycf3 | ycf3 | ycf3 | ycf3 | ycf3 | ycf3 | ycf3 |
| 38 | trnS-GGA | trnS-GGA | trnS-GGA | trnS-GGA | trnS-GGA | trnS-GGA | trnS-GGA | trnS-GGA | trnS-GGA | trnS-GGA | trnS-GGA |
| 39 | rps4 | rps4 | rps4 | rps4 | rps4 | rps4 | rps4 | rps4 | rps4 | rps4 | rps4 |
| 40 | trnT-UGU | trnT-UGU | trnT-UGU | trnT-UGU | trnT-UGU | trnT-UGU | trnT-UGU | trnT-UGU | trnT-UGU | trnT-UGU | trnT-UGU |
| 41 | trnL-UAA | trnL-UAA | trnL-UAA | trnL-UAA | trnL-UAA | trnL-UAA | trnL-UAA | trnL-UAA | trnL-UAA | trnL-UAA | trnL-UAA |
| 42 | trnF-GAA | trnF-GAA | trnF-GAA | trnF-GAA | trnF-GAA | trnF-GAA | trnF-GAA | trnF-GAA | trnF-GAA | trnF-GAA | trnF-GAA |
| 43 | ndhJ | ndhJ | ndhJ | ndhJ | ndhJ | ndhJ | ndhJ | ndhJ | ndhJ | ndhJ | ndhJ |
| 44 | ndhK | ndhK | ndhK | ndhK | ndhK | ndhK | ndhK | ndhK | ndhK | ndhK | ndhK |
| 45 | ndhC | ndhC | ndhC | ndhC | ndhC | ndhC | ndhC | ndhC | ndhC | ndhC | ndhC |
| 46 | trnV-UAC | trnV-UAC | trnV-UAC | trnV-UAC | trnV-UAC | trnV-UAC | trnV-UAC | trnV-UAC | trnV-UAC | trnV-UAC | trnV-UAC |
| 47 | trnM-CAU | trnM-CAU | trnM-CAU | trnM-CAU | trnM-CAU | trnM-CAU | trnM-CAU | trnM-CAU | trnM-CAU | trnM-CAU | trnM-CAU |
| 48 | atpE | atpE | atpE | atpE | atpE | atpE | atpE | atpE | atpE | atpE | atpE |
| 49 | atpB | atpB | atpB | atpB | atpB | atpB | atpB | atpB | atpB | atpB | atpB |
| 50 | rbcL | rbcL | rbcL | rbcL | rbcL | rbcL | rbcL | rbcL | rbcL | rbcL | rbcL |
| 51 | accD | accD | accD | accD | accD | accD | accD | accD | accD | accD | *** |
| 52 | psaI | psaI | psaI | psaI | psaI | psaI | psaI | psaI | psaI | psaI | psaI |
| 53 | ycf4 | ycf4 | ycf4 | ycf4 | ycf4 | ycf4 | ycf4 | ycf4 | ycf4 | ycf4 | ycf4 |
| 54 | cemA | cemA | cemA | cemA | cemA | cemA | cemA | cemA | cemA | cemA | cemA |
| 55 | petA | petA | petA | petA | petA | petA | petA | petA | petA | petA | petA |
| 56 | psbJ | psbJ | psbJ | psbJ | psbJ | psbJ | psbJ | psbJ | psbJ | psbJ | psbJ |
| 57 | psbL | psbL | psbL | psbL | psbL | psbL | psbL | psbL | psbL | psbL | psbL |
| 58 | psbF | psbF | psbF | psbF | psbF | psbF | psbF | psbF | psbF | psbF | psbF |
| 59 | psbE | psbE | psbE | psbE | psbE | psbE | psbE | psbE | psbE | psbE | psbE |
| 60 | petL | petL | petL | petL | petL | petL | petL | petL | petL | petL | petL |
| 61 | petG | petG | petG | petG | petG | petG | petG | petG | petG | petG | petG |
| 62 | trnW-CCA | trnW-CCA | trnW-CCA | trnW-CCA | trnW-CCA | trnW-CCA | trnW-CCA | trnW-CCA | trnW-CCA | trnW-CCA | trnW-CCA |
| 63 | trnP-UGG | trnP-UGG | trnP-UGG | trnP-UGG | trnP-UGG | trnP-UGG | trnP-UGG | trnP-UGG | trnP-UGG | trnP-UGG | trnP-UGG |
| 64 | psaJ | psaJ | psaJ | psaJ | psaJ | psaJ | psaJ | psaJ | psaJ | psaJ | psaJ |
| 65 | rpl33 | rpl33 | rpl33 | rpl33 | rpl33 | rpl33 | rpl33 | rpl33 | rpl33 | rpl33 | rpl33 |
| 66 | rps18 | rps18 | rps18 | rps18 | rps18 | rps18 | rps18 | rps18 | rps18 | rps18 | rps18 |
| 67 | rpl20 | rpl20 | rpl20 | rpl20 | rpl20 | rpl20 | rpl20 | rpl20 | rpl20 | rpl20 | rpl20 |
| 68 | clpP | clpP | clpP | clpP | clpP | clpP | clpP | clpP | clpP | clpP | clpP |
| 69 | psbB | psbB | psbB | psbB | psbB | psbB | psbB | psbB | psbB | psbB | psi_psbT |
| 70 | psbT | psbT | psbT | psbT | psbT | psbT | psbT | psbT | psbT | psbT | psbT |
| 71 | psbN | psbN | psbN | psbN | psbN | psbN | psbN | psbN | psbN | psbN | psbN |
| 72 | psbH | psbH | psbH | psbH | psbH | psbH | psbH | psbH | psbH | psbH | psbH |
| 73 | petB | petB | petB | petB | petB | petB | petB | petB | petB | petB | petB |
| 74 | petD | petD | petD | petD | petD | petD | petD | petD | petD | petD | petD |
| 75 | rpoA | rpoA | rpoA | rpoA | rpoA | rpoA | rpoA | rpoA | rpoA | rpoA | rpoA |
| 76 | rps11 | rps11 | rps11 | rps11 | rps11 | rps11 | rps11 | rps11 | rps11 | rps11 | rps11 |
| 77 | rpl36 | rpl36 | rpl36 | rpl36 | rpl36 | rpl36 | rpl36 | rpl36 | rpl36 | rpl36 | rpl36 |
| 78 | infA | infA | infA | infA | infA | infA | infA | infA | infA | infA | infA |
| 79 | rps8 | rps8 | rps8 | rps8 | rps8 | rps8 | rps8 | rps8 | rps8 | rps8 | rps8 |
| 80 | rpl14 | rpl14 | rpl14 | rpl14 | rpl14 | rpl14 | rpl14 | rpl14 | rpl14 | rpl14 | rpl14 |
| 81 | rpl16 | rpl16 | rpl16 | rpl16 | rpl16 | rpl16 | rpl16 | rpl16 | rpl16 | rpl16 | rpl16 |
| 82 | rps3 | rps3 | rps3 | rps3 | rps3 | rps3 | rps3 | rps3 | rps3 | rps3 | rps3 |
| 83 | rpl22 | rpl22 | rpl22 | rpl22 | rpl22 | rpl22 | rpl22 | rpl22 | rpl22 | rpl22 | rpl22 |
| 84 | rps19 | rps19 | rps19 | rps19 | rps19 | rps19 | rps19 | rps19 | rps19 | rps19 | rps19 |
| 85 | rpl2 | rpl2 | rpl2 | rpl2 | rpl2 | rp12 | rp12 | rpl2 | rpl2 | rpl2 | rpl2 |
| 86 | rpl23 | rpl23 | rpl23 | rpl23 | rpl23 | rpl23 | rpl23 | rpl23 | rpl23 | rpl23 | rpl23 |
| 87 | trnI-CAU | trnI-CAU | trnI-CAU | trnI-CAU | trnI-CAU | trnI-CAU | trnI-CAU | trnH-CAU | trnI-CAU | trnH-CAU | trnI-CAU |
| 88 | ycf2 | ycf2 | ycf2 | ycf2 | ycf2 | ycf2 | ycf2 | ycf2 | ycf2 | ycf2 | ycf2 |
| 89 | ycf15 | ycf15 | ycf15 | ycf15 | ycf15 | ycf15 | ycf15 | ycf15 |  | ycf15 | * |
| 90 | trnL-CAA | trnL-CAA | trnL-CAA | trnL-CAA | trnL-CAA | trnL-CAA | trnL-CAA | trnL-CAA | trnL-CAA | trnL-CAA | trnL-CAA |
| 91 | ndhB | ndhB | ndhB | ndhB | ndhB | ndhB | ndhB | ndhB | ndhB | ndhB | ndhB |
| 92 | rps7 | rps7 | rps7 | rps7 | rps7 | rps7 | rps7 | rps7 | rps7 | rps7 | rps7 |
| 93 | trnV-GAC | trnV-GAC | trnV-GAC | trnV-GAC | trnV-GAC | trnV-GAC | trnV-GAC | trnV-GAC | trnV-GAC | trnV-GAC | trnV-GAC |
| 94 | rrn16 | rrn16 | rrn16 | rrn16 | rrn16 | rrn16 | rrn16 | rrn16 | rrn16 | rrn16 | rrn16 |
| 95 | trnI-GAU | trnI-GAU | trnI-GAU | trnI-GAU | trnI-GAU | trnI-GAU | trnI-GAU | trnI-GAU | trnI-GAU | trnI-GAU | trnI-GAU |
| 96 | trnA-UGC | trnA-UGC | trnA-UGC | trnA-UGC | trnA-UGC | trnA-UGC | trnA-UGC | trnA-UGC | trnA-UGC | trnA-UGC | trnA-UGC |
| 97 | rrn23 | rrn23 | rrn23 | rrn23 | rrn23 | rrn23 | rrn23 | rrn23 | rrn23 | rrn23 | rrn23 |
| 98 | rrn4.5 | rrn4.5 | rrn4.5 | rrn4.5 | rrn4.5 | rrn4.5 | rrn4.5 | rrn4.5 | rrn4.5 | rrn4.5 | rrn4.5 |
| 99 | rrn5 | rrn5 | rrn5 | rrn5 | rrn5 | rrn5 | rrn5 | rrn5 | rrn5 | rrn5 | rrn5 |
| 100 | trnR-ACG | trnR-ACG | trnR-ACG | trnR-ACG | trnR-ACG | trnR-ACG | trnR-ACG | trnR-ACG | trnR-ACG | trnR-ACG | trnR-ACG |
| 101 | trnN-GUU | trnN-GUU | trnN-GUU | trnN-GUU | trnN-GUU | trnN-GUU | trnN-GUU | trnN-GUU | trnN-GUU | trnN-GUU | trnN-GUU |
| 102 | *ycf1/pseudo* | *ycf1/pseudo* | *ycf1/pseudo* | *ycf1/pseudo* | *ycf1/pseudo* |  |  | *ycf1/pseudo* | *ycf1/pseudo* | *ycf1/pseudo* | *ycf1* |
| 103 | *ndhF* | *ndhF* | *ndhF* | *ndhF* | *ndhF* | *ndhF* | *ndhF* | *ndhF* | *ndhF* | *ndhF* | *ndhF* |
| 104 | *rpl32* | *rpl32* | *rpl32* | *rpl32* | *rpl32* | *rpl32* | *rpl32* | *rpl32* | *rpl32* | *rpl32* | *rpl32* |
| 105 | *trnL-UAG* | *trnL-UAG* | *trnL-UAG* | *trnL-UAG* | *trnL-UAG* | *trnL-UAG* | *trnL-UAG* | *trnL-UAG* | *trnL-UAG* | *trnL-UAG* | *trnL-UAG* |
| 106 | *ccsA* | *ccsA* | *ccsA* | *ccsA* | *ccsA* | *ccsA* | *ccsA* | *ccsA* | *ccsA* | *ccsA* | *ccsA* |
| 107 | *ndhD* | *ndhD* | *ndhD* | *ndhD* | *ndhD* | *ndhD* | *ndhD* | *ndhD* | *ndhD* | *ndhD* | *ndhD* |
| 108 | *psaC* | *psaC* | *psaC* | *psaC* | *psaC* | *psaC* | *psaC* | *psaC* | *psaC* | *psaC* | *psaC* |
| 109 | *ndhE* | *ndhE* | *ndhE* | *ndhE* | *ndhE* | *ndhE* | *ndhE* | *ndhE* | *ndhE* | *ndhE* | *ndhE* |
| 110 | *ndhG* | *ndhG* | *ndhG* | *ndhG* | *ndhG* | *ndhG* | *ndhG* | *ndhG* | *ndhG* | *ndhG* | *ndhG* |
| 111 | *ndhI* | *ndhI* | *ndhI* | *ndhI* | *ndhI* | *ndhI* | *ndhI* | *ndhI* | *ndhI* | *ndhI* | *ndhI* |
| 112 | *ndhA* | *ndhA* | *ndhA* | *ndhA* | *ndhA* | *ndhA* | *ndhA* | *ndhA* | *ndhA* | *ndhA* | *ndhA* |
| 113 | *ndhH* | *ndhH* | *ndhH* | *ndhH* | *ndhH* | *ndhH* | *ndhH* | *ndhH* | *ndhH* | *ndhH* | *ndhH* |
| 114 | *rps15* | *rps15* | *rps15* | *rps15* | *rps15* | *rps15* | *rps15* | *rps15* | *rps15* | *rps15* | *rps15* |
| 115 | *ycf1* | *ycf1* | *ycf1* | *ycf1* | *ycf1* | *ycf1* | *ycf1* | *ycf1* | *ycf1* | *ycf1* | *ycf1* |
| 116 | trnN-GUU | trnN-GUU | trnN-GUU | trnN-GUU | trnN-GUU | trnN-GUU | trnN-GUU | trnN-GUU | trnN-GUU | trnN-GUU | trnN-GUU |
| 117 | trnR-ACG | trnR-ACG | trnR-ACG | trnR-ACG | trnR-ACG | trnR-ACG | trnR-ACG | trnR-ACG | trnR-ACG | trnR-ACG | trnR-ACG |
| 118 | rrn5 | rrn5 | rrn5 | rrn5 | rrn5 | rrn5 | rrn5 | rrn5 | rrn5 | rrn5 | rrn5 |
| 119 | rrn4.5 | rrn4.5 | rrn4.5 | rrn4.5 | rrn4.5 | rrn4.5 | rrn4.5 | rrn4.5 | rrn4.5 | rrn4.5 | rrn4.5 |
| 120 | rrn23 | rrn23 | rrn23 | rrn23 | rrn23 | rrn23 | rrn23 | rrn23 | rrn23 | rrn23 | rrn23 |
| 121 | trnA-UGC | trnA-UGC | trnA-UGC | trnA-UGC | trnA-UGC | trnA-UGC | trnA-UGC | trnA-UGC | trnA-UGC | trnA-UGC | trnA-UGC |
| 122 | trnI-GAU | trnI-GAU | trnI-GAU | trnI-GAU | trnI-GAU | trnI-GAU | trnI-GAU | trnI-GAU | trnI-GAU | trnI-GAU | trnI-GAU |
| 123 | rrn16 | rrn16 | rrn16 | rrn16 | rrn16 | rrn16 | rrn16 | rrn16 | rrn16 | rrn16 | rrn16 |
| 124 | trnV-GAC | trnV-GAC | trnV-GAC | trnV-GAC | trnV-GAC | trnV-GAC | trnV-GAC | trnV-GAC | trnV-GAC | trnV-GAC | trnV-GAC |
| 125 | rps7 | rps7 | rps7 | rps7 | rps7 | rps7 | rps7 | rps7 | rps7 | rps7 | rps7 |
| 126 | ndhB | ndhB | ndhB | ndhB | ndhB | ndhB | ndhB | ndhB | ndhB | ndhB | ndhB |
| 127 | trnL-CAA | trnL-CAA | trnL-CAA | trnL-CAA | trnL-CAA | trnL-CAA | trnL-CAA | trnL-CAA | trnL-CAA | trnL-CAA | trnL-CAA |
| 128 | ycf15 | ycf15 | ycf15 | ycf15 | ycf15 | ycf15 | ycf15 | ycf15 |  | ycf15 | * |
| 129 | ycf2 | ycf2 | ycf2 | ycf2 | ycf2 | ycf2 | ycf2 | ycf2 | ycf2 | ycf2 | ycf2 |
| 130 | trnI-CAU | trnI-CAU | trnI-CAU | trnI-CAU | trnI-CAU | trnI-CAU | trnI-CAU | trnH-CAU | trnI-CAU | trnH-CAU | trnI-CAU |
| 131 | rpl23 | rpl23 | rpl23 | rpl23 | rpl23 | rpl23 | rpl23 | rpl23 | rpl23 | rpl23 | rpl23 |
| 132 | rpl2 | rpl2 | rpl2 | rpl2 | rpl2 | rp12 | rp12 | rpl2 | rpl2 | rpl2 | rpl2 |
| 133 | rps19/pseudo | rps19/pseudo | rps19/pseudo | rps19/pseudo | rps19/pseudo |  |  | rps19/pseudo | rps19/pseudo | rps19/pseudo | * |

^￥^ indicates *Aralia undulate* for KC456163, *Brassaiopsis hainla* for KC456164, *Metapanax delavayi* for KC456165, *Schefflera delavayi* for KC456166, *Kalopanax septemlobus* for KC456167, *Panax ginseng* *ermaya* for KC686332, Panax ginseng *gaolishen* for KC686333, *Panax ginseng* for AY582139, *Camellia sinensis* for KC143082, *Anthriscus cerafolium* for GU456628, *Eleutherococcus sentocosus* for JN637765; * indicates genes substituted and missing and/or non-identified in *B. falcatum*.

**Table S2.** The result of tandem repeat sequences detected in the *B. falcatum* chloroplast genome sequence.

| **No.** | **Locus** | **Consensus Size** | **Copy Number** | **Consensus (5**′ → **3**′**)** |
| --- | --- | --- | --- | --- |
| 1 | 9407–9444 | 19 | 2 | ATTATAGAATATAACATCA |
| 2 | 9634–9647 | 7 | 2 | TTTTTAT |
| 3 | 10226–10241 | 8 | 2 | TATTATTT |
| 4 | 13924–13941 | 9 | 2 | ATTTATATA |
| 5 | 14766–14783 | 9 | 2 | ATTCTTTGA |
| 6 | 23495–23508 | 7 | 2 | TATTCAA |
| 7 | 28356–28375 | 10 | 2 | TGCCTATTTC |
| 8 | 28835–28850 | 8 | 2 | AATTAATA |
| 9 | 29391–29424 | 17 | 2 | TATATATAATATATAGA |
| 10 | 30070–30083 | 7 | 2 | AATTTTA |
| 11 | 32337–32354 | 9 | 2 | ATTAATAAA |
| 12 | 32737–32846 | 55 | 2 | ATTCAAGAATAAGACACGAGCCACTACGAAGTTACTGCATGGACTTAAGTATATA |
| 13 | 33524–33541 | 9 | 2 | CTTTGATTA |
| 14 | 43212–43227 | 8 | 2 | ATATTTAT |
| 15 | 43548–43565 | 9 | 2 | ATATACTAA |
| 16 | 46719–46730 | 6 | 2 | ATATAG |
| 17 | 46751–46765 | 6 | 2 | ATATAG |
| 18 | 46896–46917 | 11 | 2 | CTGAAAGGAAA |
| 19 | 49794–49811 | 6 | 3 | GGGAAT |
| 20 | 50156–50179 | 12 | 2 | CCATAATACAAA |
| 21 | 51535–51550 | 8 | 2 | TTCTTTTT |
| 22 | 52323–52334 | 6 | 2 | TATCTT |
| 23 | 53115–53126 | 6 | 2 | ATTAGG |
| 24 | 58976–58991 | 8 | 2 | TATTTAGA |
| 25 | 60750–60765 | 8 | 2 | TAAGTTAA |
| 26 | 61081–61102 | 11 | 2 | TACATATCTTT |
| 27 | 61357–61368 | 6 | 2 | GGCAAT |
| 28 | 65508–65523 | 8 | 2 | ATAAAAAT |
| 29 | 67946–67961 | 8 | 2 | ATTAAAAT |
| 30 | 68454–68467 | 7 | 2 | AATAGTA |
| 31 | 69192–69205 | 7 | 2 | TTTTTTA |
| 32 | 69322–69335 | 7 | 2 | AAATCAA |
| 33 | 71944–71961 | 9 | 2 | ATAAAGAAT |
| 34 | 73026–73043 | 9 | 2 | TAATATATA |
| 35 | 77251–77278 | 14 | 2 | TTTTTCTTGTCTCC |
| 36 | 78701–78716 | 8 | 2 | ACTTAATC |
| 37 | 80108–80119 | 6 | 2 | CATTCC |
| 38 | 84223–84254 | 16 | 2 | TTATTAGTTTATACTA |
| 39 | 85581–85598 | 9 | 2 | TTTATCTTC |
| 40 | 85644–85663 | 2 | 10 | AT |
| 41 | 88157–88180 | 12 | 2 | ATTGAGAGAGAT |
| 42 | 89981–89998 | 9 | 2 | GGAACATTT |
| 43 | 94568–94585 | 9 | 2 | GTATGGATG |
| 44 | 95627–95640 | 7 | 2 | AGATTAT |
| 45 | 112274–112291 | 9 | 2 | AAAAAAAAG |
| 46 | 114643–114658 | 8 | 2 | TTCAAATA |
| 47 | 114751–114774 | 12 | 2 | GAAATTTATTAA |
| 48 | 114820–114835 | 8 | 2 | ACATTAAA |
| 49 | 119280–119291 | 6 | 2 | ATACAT |
| 50 | 121624–121637 | 7 | 2 | TTTCCAA |
| 51 | 123501–123516 | 8 | 2 | TAAAAAAT |
| 52 | 146261–146274 | 7 | 2 | TATAATC |
| 53 | 147312–147329 | 9 | 2 | CATACCATC |
| 54 | 151904–151921 | 9 | 2 | AAATGTTCC |
| 55 | 153722–153745 | 12 | 2 | ATCTCTCTCAAT |
